# Design of experiment-oriented development of solvent-free mixed micellar chromatographic method for concomitant determination of metronidazole and ciprofloxacin hydrochloride

**DOI:** 10.1038/s41598-023-44498-5

**Published:** 2023-10-13

**Authors:** Sherin F. Hammad, Ahmed A. Habib, Amira H. Kamal, Safa M. Megahed

**Affiliations:** Department of Pharmaceutical Analytical Chemistry, Faculty of Pharmacy, Tanta University, Tanta, Egypt

**Keywords:** Chemistry, Analytical chemistry

## Abstract

A green, fast and robust solvent-free chromatographic method has been developed for concomitant analysis of ciprofloxacin HCl and metronidazole in bulk powder as well as in dosage form using levofloxacin as internal standard (I.S.). Two different designs including fractional factorial (FFD) and Box–Behnken (BBD) designs were implemented for screening and optimization steps, respectively. The optimum chromatographic separation was accomplished using mobile phase composed of 0.13 M sodium dodecyl sulfate and 0.02 M Birij-35 solution adjusted to pH 2.5 using phosphoric acid at a flow rate of 1.3 mL/min and column oven temperature of 40 °C. Chromatographic analysis was performed on X-Bridge (150 mm × 4.6 mm, 5 μm) column with UV detection at 280 nm. A linear response was acquired over the range of 0.4–50 μg/mL for both drugs. The developed method was applied for quantitation of cited drugs in commercially available tablet with mean percent recovery ± SD of 99.45 ± 0.72 and 100.13 ± 0.81 for metronidazole and ciprofloxacin respectively. The method was proven to be green as evaluated by three greenness assessment tools. The run time was 8 min, thus saving time and reagent.

## Introduction

Nowadays, there is growing interest in development of environment-friendly analytical methods that cause less harm to human and environment. There are different ways for greening chromatographic techniques. For instance, replacing the highly toxic solvents with more ecofriendly ones and reducing the amount of organic solvents used. Another way is to totally eliminate the use of organic solvents in the mobile phase, and use aqueous solutions of surfactants at concentration above its critical micelle concentration instead. The later technique is called micellar chromatography. The use of surfactants such as sodium dodecyl sulfate (SDS) usually requires addition of small amount of organic solvent to increase elution power and improve separation of peaks. However, the addition of another non-ionic surfactant like Birij-35 allows elution of different compounds without adding any organic solvents^1^. The technique that involves using mixture of two surfactants is called mixed micellar chromatography. It has a great advantage of being green and environment friendly method. It has been implemented for determination of several drugs^2–5^.

Different tools have been proposed and applied for assessment of analytical procedures greenness^6–9^. Analytical Eco-Scale assesses greenness depending on penalty points assigned for each reagent amount and type, the energy consumption by instruments and the waste treatment manner^10^. A new tool has been introduced for greenness evaluation of analytical procedures based on principles derived from the twelve principles of green analytical chemistry called AGREE^11^. Another recently introduced greenness assessment tool is ComplexGAPI which represent method greenness in the form of colored pictograms^12^.

Recently, implementation of principles of Quality by Design (QbD) in various pharmaceutical aspects is gaining interest. The implementation of QbD paradigm to analytical procedure development emphasizes the concept of establishing quality during method development, rather than testing method’s quality after development. QbD paradigm follows a systematic path in method development thus ensures method’s robustness.

First, the goal of the method to be developed, which is called Analytical Target Profile (ATP), is defined and then CMPs (critical method parameters) that have impact on CQAs (critical quality attributes) are assigned. 

Various experimental designs have been performed during validation, development, and optimization of different analytical procedures^13–16^. Among available designs fractional factorial design is commonly applied in screening of large number of factors with few experimental runs. Box Behnken Design (BBD) is used to attain optimum conditions by studying the parameters at three levels with a fewer experiments compared to other three level designs such as central composite and three level full factorial designs^17^. 

**Figure 1:**
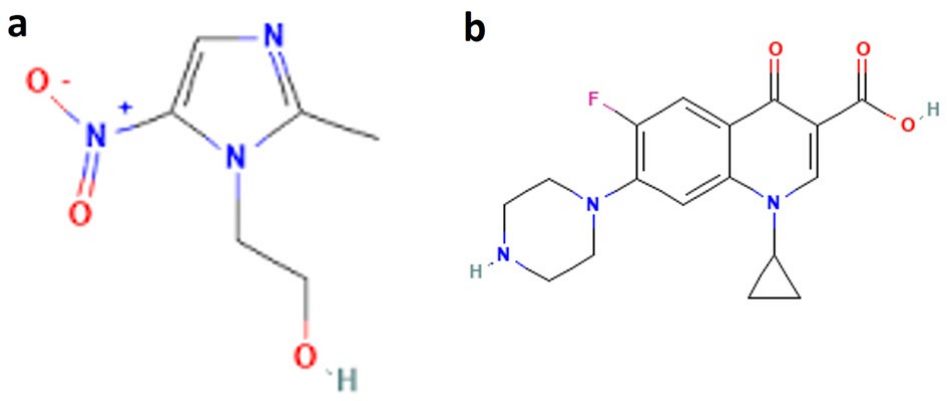
Chemical structure of (**a**) MTR, and (**b**) CIP.

Ciprofloxacin hydrochloride (CIP HCl) is one of fluoro-quinolone antibacterial agent that has effect on different bacterial strains that cause urinary tract, respiratory, and abdominal infections. Metronidazole (MTR) belongs to nitro-imidazole antimicrobial drugs specific for protozoa and anaerobic bacteria. The dosage form containing combination of MTR and CIP is commonly used for broad spectrum of infections caused by anaerobic bacteria^18^. The structure of cited drugs is shown in Fig. 1. A comprehensive review of literature reveals that different methods have been reported for concomitant analysis of CIP HCl and MTR including UV spectrophotometric methods^19,20^, electrochemical sensor^21^, and chromatographic methods^22–27^. However, all the reported chromatographic methods involved the use of acetonitrile or methanol as organic modifier which cause harm to health and environment. Therefore, there is a need for developing green eco-friendly chromatographic method for rapid determination of cited drugs that can replace the reported methods in routine analysis.

The aim of the current work was to develop green micellar chromatographic method for concomitant analysis of MTR and CIP HCl, by eliminating the use of harmful organic solvents as well as minimizing run time to reduce cost and energy consumption. It involved implementation of designs of experiment for screening and optimization of all chromatographic factors.

New trend have evolved for combining green analytical chemistry and QbD concepts into a single technique in order to increase its robustness and sustainability. As a result, analysts are trying to make a compromise between development of sensitive and selective analytical methods while applying more eco-friendly and energy efficient techniques^28–31^.

The greenness of the method was incorporated in the development of the method from the first step by assigning Analytical Eco-Scale as response to be optimized throughout method development. The novelty of the developed method depends on being the first green solvent free chromatographic method for concomitant assay of CIP HCl and MTR.

## Materials and methods

### Materials and reagents

All chemicals utilized throughout the present work were of analytical grade. CIP HCl powder was kindly gifted by Pharmed H (Elsadat City, Egypt) certified to contain 100.17% CIP HCl. MTR powder (100.10 %) was provided as a gift from Sigma Tec Pharmaceutical Industries, 6th October, Giza, Egypt.

Sodium dodecyl sulphate (Belami fine chemicals, India), Birij-35 (Fischer scientific, Germany), orthophosphoric acid (Ridel-de Haën, Germany) were used. Milli-Q water purifcation system (USA) was used to get deionized water.

### Pharmaceutical dosage form

Ciprodiazole tablets (Batch number AKE2558, Minapharm, Egypt) were bought from local pharmacy. Each single tablet is labelled to contain 524.35 mg CIP HCl monohydrate equivalent to 500 mg CIP HCl and 500 mg MTR.

### Instrument and software

Chromatographic analysis was accomplished using Agilent Technologies 1260 Infinity HPLC (USA) composed of a quaternary pump (G1311C), auto-sampler (G1329B), thermostated column compartment (G13b16A), and UV detector (G1314F). A Dell computer was connected to the HPLC instrument, provided with Agilent software (OpenLAB CDS Chemstation). Experimental design analysis including matrix of fractional factorial (FFD) and Box–Behnken (BBD) designs were executed using Design Expert software (Stat_Ease Inc. Minneapolis, USA); version 11.1.2.0.

### Chromatographic conditions

Chromatographic analysis was accomplished on X-Bridge column (150 mm × 4.6 mm, 5 μm) using mixture of 0.13 M SDS and 0.02 M Birij-35 solution adjusted to pH 2.5 with ortho-phosphoric acid as a mobile phase pumped at 1.3 mL/min flow rate. Column temperature was kept at 40 °C throughout analysis. The detection was performed at 280 nm using UV detector.

### Preparation of stock and working standard solutions

MTR stock solution that has a concentration of 500 µg/mL was prepared by dissolving 25 mg of MTR powder in distilled water in 50-mL volumetric flask. Stock solution of CIP HCl was prepared similarly by dissolving 25 mg of CIP HCl powder in distilled water in 50-mL volumetric flask to make a solution of 500 µg/mL. Working solution containing 100 µg/mL of each drug was prepared separately by taking a volume of 20 mL from the stock solution of each drug and then diluting it to 100 mL with water in a 100-mL volumetric flask. Stock solution of levofloxacin (LVF) internal standard (I.S.) was prepared by weighing 25 mg of LVF, then dissolving the powder in 250 mL distilled water to get 100 µg/mL solution.

### Construction of calibration curves

Into a group of 10-mL volumetric flasks, aliquots of MTR and CIP HCl working standard solutions were quantitatively transferred; one mL of LVF stock solution was added; then appropriate dilution with the mobile phase was carried out to obtain solutions in the concentration range of 0.5–50 μg/mL for both MTR and CIP HCl and 10 μg/mL of LVF (I.S.). Portions of each solution (injection volume of 20 µL) were injected in triplicates under the mentioned chromatographic conditions.

### Analysis of tablet dosage form

Ten Ciprodiazole tablets were weighed and grinded into fine powder in a mortar. A quantity equivalent to 50 mg MTR and 50 mg CIP HCl was transferred into 100-mL measuring flask and 50 mL distilled water was added, then the flask was sonicated for 20 min. After sonication, distilled water was added to fill the flask to the specified mark. This solution was then filtered through a 0.45 µm membrane filter, the first portion of the filtrate was discarded, and then 2 mL of the filtrate was transferred to a 10-mL volumetric flask, 1 mL of LVF stock solution was added, and then completed to the specified mark with mobile phase to get a solution containing 25 µg/mL of MTR and CIP HCl and 10 µg/mL LVF (I.S.). On a further five portions of the filtrate, these steps were repeated. The regression equation was used to determine the concentration of each drug in the tablet.

## Results and discussion

### Method development and optimization

Quality by Design paradigm was implemented throughout the development of the method. First, ATP (Analytical target profile), which is the purpose of the method is assigned. The purpose was development of eco-friendly solvent free chromatographic method for concomitant analysis of MTR and CIP HCl within short analysis time. Then, CQAs (critical quality attributes) are defined including rapid analysis, good peak shape, increased number of theoretical plates and the method greenness. The method greenness was taken as one of the responses to be optimized from the first step in screening phase in order to get the best chromatographic conditions not only regarding analytical performance but regarding greenness as well. Then, preliminary experiments were carried out to identify CMPs (critical method parameters) which could affect CQAs.

### Screening of parameters

The traditional trial and error method for optimization of chromatographic conditions is tedious due to large number of factors to be studied. Fractional factorial (FFD) design was selected for screening phase as it is the most convenient design for studying a large number of factors. FFD was executed for screening of five factors which are SDS concentration, Birij-35 concentration, pH of the mobile phase, triethylamine concentration, and column temperature. A number of twelve experiments were executed as presented in design matrix in supplementary material (Table S1). Different responses including resolution between MTR and CIP peaks, tailing of peaks, number of theoretical plates for each peak (N), capacity factor of MTR peak (k'), run time, and Analytical Eco-Scale were recorded. Number of theoretical plates was taken as one of the responses during the screening and optimization phases due to poor column efficiency associated with micellar chromatography because of slow mass transfer from the stationary phase^32^. So, there is a need to select the chromatographic conditions that maximize N. Analytical Eco-Scale was calculated for each experiment as indicative tools for method greenness.

### Fractional factorial design results

From FFD result, coefficients were obtained and statistical analysis was performed to explain the relationship between experimental factors and different responses for CIP HCl and MTR chromatographic analysis as shown in supplementary material (Table S3 and Table S4).

**Figure 2:**
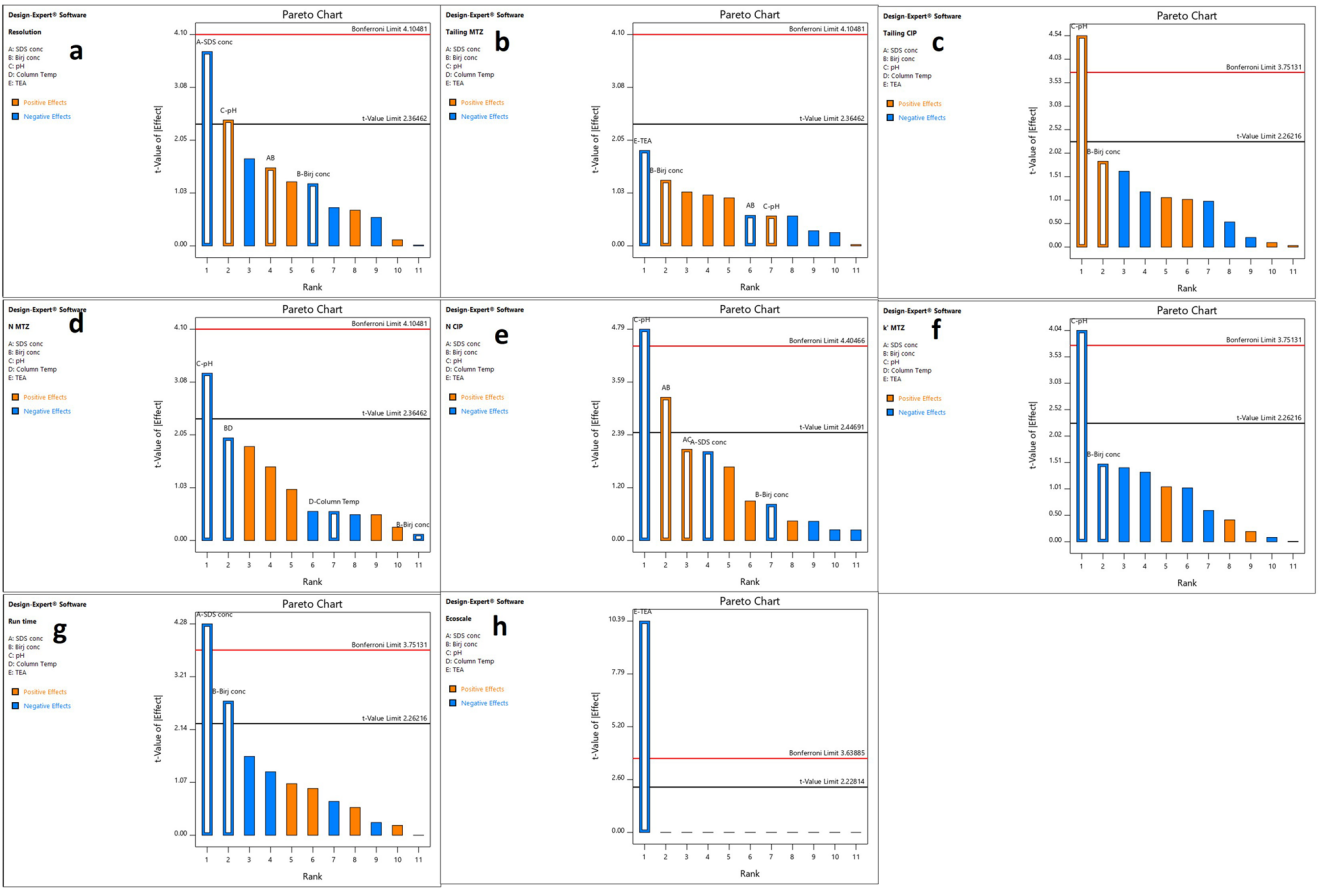
Pareto charts of the effects on the chromatographic responses: (**a**) resolution, (**b**) tailing of MTR, (**c**) tailing of CIP, (**d**) N of MTR, (**e**) N of CIP, (**f**) k’ of MTR, (**g**) run time, and (**h**) analytical ecoscale score.

The most statistically significant factors that greatly affect different responses were identified using pareto charts. Significant factors are those which exceed t-limit line. Figure 2 presents Pareto charts which show that pH of the mobile phase has significant effect on resolution between peaks, tailing of BNX peak, tailing of CIP peak, N of both CIP and MTR peaks and k' of MTR peak meanwhile SDS concentration significantly affects resolution and run time. Birij-35 concentration also has significant effect on run time. Interaction between concentration of SDS and Birij-35 significantly affects N of CIP peak. Column temperature did not have any significant impact on any response. Triethylamine had negative effect on Analytical Eco-Scale and it did not affect any other CQA, therefore it was removed from mobile phase to improve greenness of the method. From the pareto charts results, three factors which have the greatest significance were selected for further optimization including pH of the mobile phase, SDS concentration and Birij-35 concentration. The column temperature was kept at 40 °C.

### Optimization using Box Behnken design

For optimization of the crucial factors, Box Behnken (BBD) design was applied as it requires less number of experimental runs to get quadratic model compared to other experimental designs such as Central Composite (CCD) Design. A number of 15 experimental runs were executed and responses were recorded as presented in supplementary material (Table S2).

### Box Behnken design results

The data obtained from the fifteen runs was analyzed by the aid of Design Expert software, and various plots were generated. 3D response surface plot is very informative about the dual significant interaction impacts^33,34^. It shows how the response changes depending on the factors values and are used to find the optimal conditions^35,36^. A color code is associated with each plot to allow prediction of the response’s value. Blue color represents the low value whereas red color indicates greater value of a response. 3D surface plots in supplementary material (Figure S1 and S2) illustrated the effect of SDS and Birij-35 concentrations on different responses. From these plots, acceptable middle values of N and k' of MTR were obtained at all concentration range under study. Increasing SDS concentration is associated with decrease resolution, run time, N CIP, and symmetry of MTR peak. Decreasing Birij-35 concentration results in decrease tailing of CIP peak.

**Figure 3:**
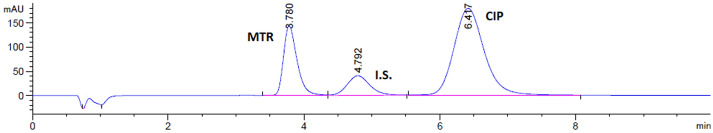
HPLC Chromatogram of 25 µg/mL MTR and 25 µg/mL CIP using 10 µg/mL LVF (I.S.).

Desirability plot helped to identify the optimum chromatographic conditions as shown in supplementary material (Figure S3). The optimum conditions involved the use of mobile phase consisting of a mixture of 0.13 M SDS and 0.02 M Birij-35 solution and pH adjusted to 2.5 pumped at 1.3 mL/min flow rate and column oven temperature of 40 °C. Chromatogram of a binary mixture containing 25 µg/mL MTR and 25 µg/mL CIP HCl using 10 µg/mL LVF as internal standard (I.S.) under the previously mentioned chromatographic conditions is shown in Fig. 3.

### Design space deduction

The design space provides the range of interactions between crucial factors and their impact on different responses that had been studied during optimization step. It assures developed method’s quality. Regions that are shaded out in grey represents the range of factors where the specifications of a given response were not met, while the yellow region represents a favorable region at which desired responses are full filled as shown in supplementary material (Figure S4). In other words, within the yellow regions in design space the method is robust and the quality of the proposed method will not be affected by changes made within this region.

### Method validation

The developed analytical procedure was validated in compliance with the ICH guidelines^37^.

#### Linearity and range

The linearity of MTR and SPR was confirmed by construction of the calibration curves in the range of 0.5–50 µg/mL for both drugs. Calibration curves were acquired by plotting ratios of peak area (peak area of MTR or CIP HCl/peak area of I.S.) versus concentration of each drug. The correlation coefficients values (0.9998 for MTR and 0.9997 for CIP HCl) indicated the good linearity. Linearity regression parameters for MTR and CIP HCl using the proposed HPLC method are presented in Table 1.

**Table 1:** Linearity regression parameters for MTR and CIP HCl using the proposed HPLC method.

Parameter	MTR	CIP HCl
Linearity range (µg/mL)	0.5–50	0.5–50
slope	0.06	0.19
SE of slope	0.0003	0.0012
Intercept	0.03	0.04
SE of Intercept	0.007	0.03
Correlation coefficient (r)	0.9998	0.9997
SE of estimation	0.015	0.07

*SE* standard error.

#### Limits of quantitation (LOQ) and detection (LOD)

The ICH (Q2 (R1)) guidelines were implemented for the determination of LOQ and LOD based on the standard deviation of the blank response and the slope of the calibration curve. For MTR, the values of LOD and LOQ were 0.12 and 0.37 µg/mL, while for CIP HCl, they were 0.11 and 0.33 µg/mL.

#### Accuracy and precision

Accuracy of the developed method was assessed by calculating the mean percent recoveries of triplicate determination for MTR and CIP HCl at three concentrations within the linearity range as shown in Table 2. The good % recovery values indicate the accuracy of the developed method. Repeatability and intermediate precision (intraday and inter-day precisions) were assessed by determining the values of the standard deviation and % relative standard deviation for triplicate assays of three concentrations of MTR and CIP HCl within the linearity range in the same day and on three different days respectively. The values of % RSD did not exceed 2% as shown in Table 3 which confirmed the precision of the proposed method.

**Table 2:** Evaluation of accuracy for the determination of MTR and CIP HCl.

Drug	Conc. taken (µg/mL)	Conc. found (µg/mL)	% Recovery	Mean % recovery ± SD
MTR	10	9.86	98.60	99.10 ± 0.78
	25	24.99	99.99	
	50	49.37	98.74	
CIP HCl	10	10.06	100.58	100.23 ± 0.45
	25	25.10	100.39	
	50	49.87	99.73

**Table 3:** Evaluation of the precision of the proposed HPLC method for the determination of MTR and CIP HCl.

Drug	Intra day			Inter day		
	Conc. taken (µg/mL)	Conc. found (µg/mL)	%RSD	Conc. taken (µg/mL)	Conc. found (µg/mL)	%RSD
MTR	10	9.98	1.04	10	9.79	0.95
		9.80			9.64	
		9.81			9.81	
	25	25.12	0.51	25	24.87	0.76
		24.87			25.15	
		25.01			24.78	
	50	49.72	0.62	50	49.72	0.48
		49.24			50.08	
		49.15			49.63	
CIP HCl	10	10.12	0.86	10	9.71	1.05
		10.09			9.60	
		9.96			9.81	
	25	25.18	0.71	25	25.08	0.64
		24.89			24.87	
		25.22			25.18	
	50	49.87	0.55	50	49.09	0.91
		49.59			48.86	
		50.14			49.72	

#### Robustness

Robustness of the developed methodology was evaluated by making small variations within its optimized conditions. The parameters which were examined in the robustness testing included the change in pH (2.5 ± 0.1), concentration of SDS (0.13 ± 0.005 M), concentration of Birij-35 (0.02 ± 0.005 M), and column temperature (40 ± 2) °C. RSD values were found to be less than 2% indicating robustness of the method as shown in supplementary material (Table S5).

### Application to the tablet formulation

The developed method was implemented for the quantitation of MTR and CIP HCl in their commercially available Ciprodiazole tablet without interference from excipients. Satisfactory results were obtained for both drugs as shown in supplementary material (Table S6). The results of the developed method were compared with that of the reference method^24^. According to the findings of the students' t-test and F-test, which are displayed in the shown in supplementary material (Table S7), there was no significant difference between the adopted method and the reference one. The chromatogram of Ciprodiazole tablet is shown in supplementary material (Figure S5).

### Greenness assessment of the developed method

The proposed chromatographic method’s greenness was proved by the high value of calculated Analytical Eco-Scale score (97) as shown in supplementary material (Table S8). From the total of 100 points, penalty points are subtracted for each negative effect on the environment, such as hazardous chemicals used, waste generation and high energy consumption. The method was also proved to be green as evaluated by other two greenness assessment tools; ComplexGAPI and AGREE score. The application of ComplexGAPI is based on simple free software. It assigns the greenness of each step of an analytical methodology using a pictogram and color scale with three levels of assessment corresponding to different levels of impact on the environment, with low, medium and high levels corresponding to green, yellow and red, respectively. On the other hand, The AGREE software generates a clock-like outline with numbers 1–12 around the edge, indicating the 12 principles of green analytical chemistry.

The proposed method was compared to three reported methods and found to be much better than them regarding greenness as shown in Table 4.

**Table 4:** Comparison between the proposed HPLC method and the reported ones regarding greenness.

Mobile phase	Reported method^24^	Reported method^22^	Reported method^26^	Reported method^27^	Proposed method
	Acetonitrile: sodium phosphate buffer (pH 3.3) (20:80,v/v)	Monobasic potassium phosphate (pH 3.5): acetonitrile (80: 20, v/v) containing triethylamine (7.5 mM)	Methanol: potassium phosphate buffer (60:40 ,v/v)	Methanol: aqueous 0.05% triethylamine (25:75 v/v)	Mixture of 0.13 M SDS and 0.02 M Birij-35 solution adjusted to pH 2.5 using phosphoric acid
Analytical Eco-Scale	84	76	82	74	97
AGREE					
ComplexGAPI					

## Conclusion

A green, simple, and rapid micellar solvent free chromatographic method was developed for the concomitant determination of MTR and CIP HCl in their bulk and tablets within eight minutes. Experimental design paradigm was implemented throughout the development of the method; fractional factorial design for screening and Box–Behnken design for optimization. Combining experimental designs with green analytical chemistry results in the development of a design space that ensures the method's quality and improves its robustness. The developed method was successfully applied for quantitation of cited drugs in commercially available tablet with mean percent recovery ± SD of 99.45 ± 0.72 and 100.13 ± 0.81 for MTR and CIP HCl respectively. Being eco-friendly and fast, the adopted method can replace the reported HPLC methods for analysis of both drugs in routine analysis.

### Supplementary Information

Supplementary Information.

## Data Availability

The datasets generated and/or analyzed during this study are included in the article and supplementary material. More data are available from the corresponding author on reasonable request.
