# Supplementary material for: Design of experiment-oriented development of solvent-free mixed micellar chromatographic method for concomitant determination of metronidazole and ciprofloxacin hydrochloride
Source: Sci Rep. 2023 Oct 13;13:17352. doi: 10.1038/s41598-023-44498-5 (PMC10575926; doi:10.1038/s41598-023-44498-5)
Supplement: Supplementary file 1 — Supplementary Information. [file 41598_2023_44498_MOESM1_ESM.docx]

**Design of Experiment-Oriented Development of Solvent-Free Mixed Micellar Chromatographic Method for Concomitant Determination of Metronidazole and Ciprofloxacin Hydrochloride**

**Sherin F Hammad^1^, Ahmed A Habib^1^, Amira H. Kamal^1^, and Safa M Megahed^1^***

^1^ Department of pharmaceutical analytical chemistry, Faculty of Pharmacy, Tanta University, Tanta, Egypt.

*Corresponding author

e-mail: [safa.megahed@gmail.com](mailto:safa.megahed@gmail.com), safa.megahed.pharm.edu.tanta.eg

Supplementary material

**Figure Caption:**

**Supplementary Fig. S1:** Three dimensional plot of the effects on the chromatographic responses: resolution, tailing MTR, tailing CIP, and N MTR.

**Supplementary Fig. S2:** Three dimensional plot of the effects on the chromatographic responses: N CIP, run time, and k’ MTR.

**Supplementary Fig. S3:** Desirability plot showing optimum chromatographic conditions.

**Supplementary Fig. S4:** Design space (overlay plot) for different chromatographic conditions.

**Supplementary Fig. S5:** Chromatogram of Ciprodiazole tablet solution containing 25 µg/mL MTR and 25 µg/mL CIP HCl and 10 µg/mL LVF I.S. using the proposed HPLC method.


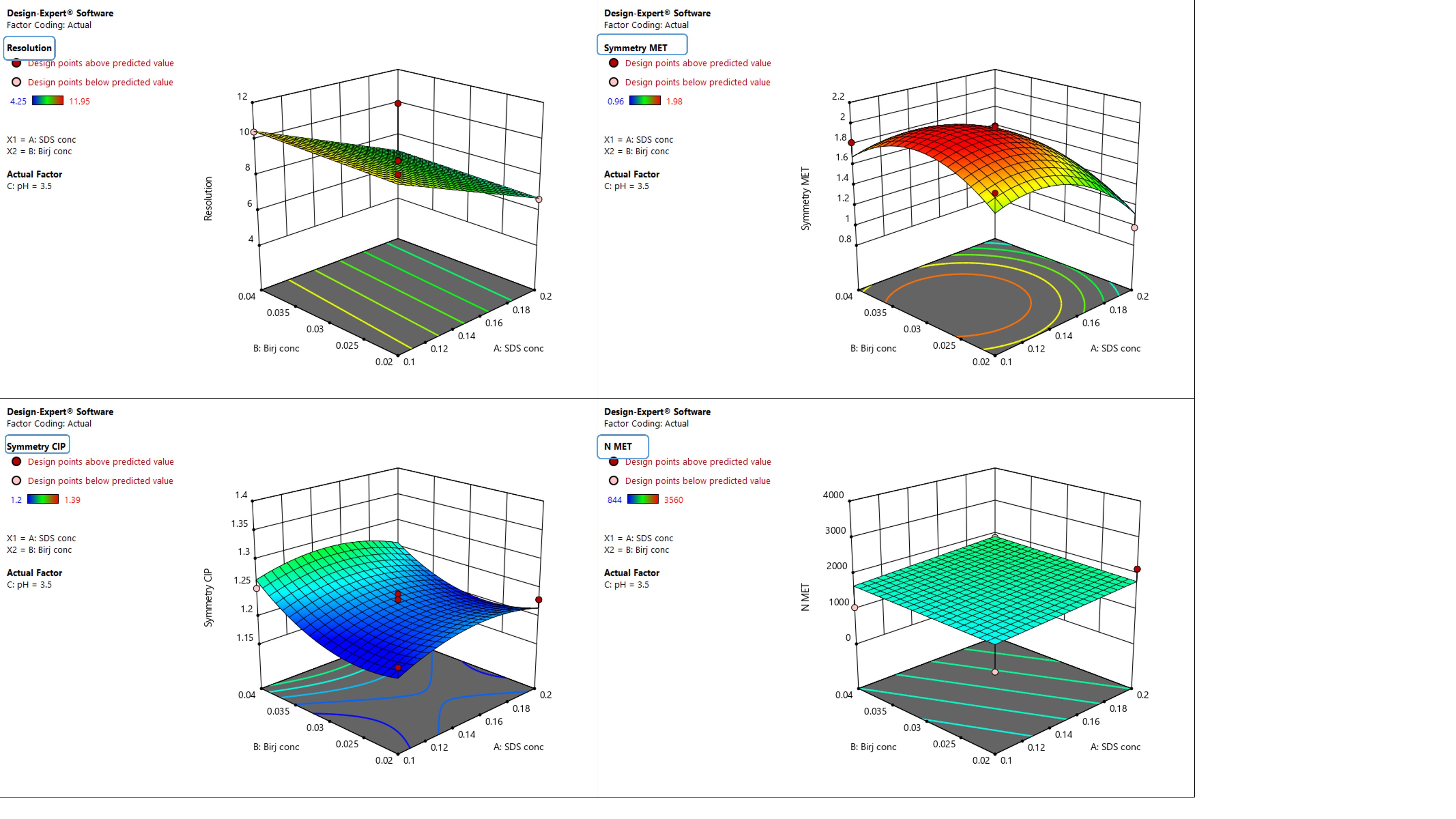


**Supplementary Fig. S1**


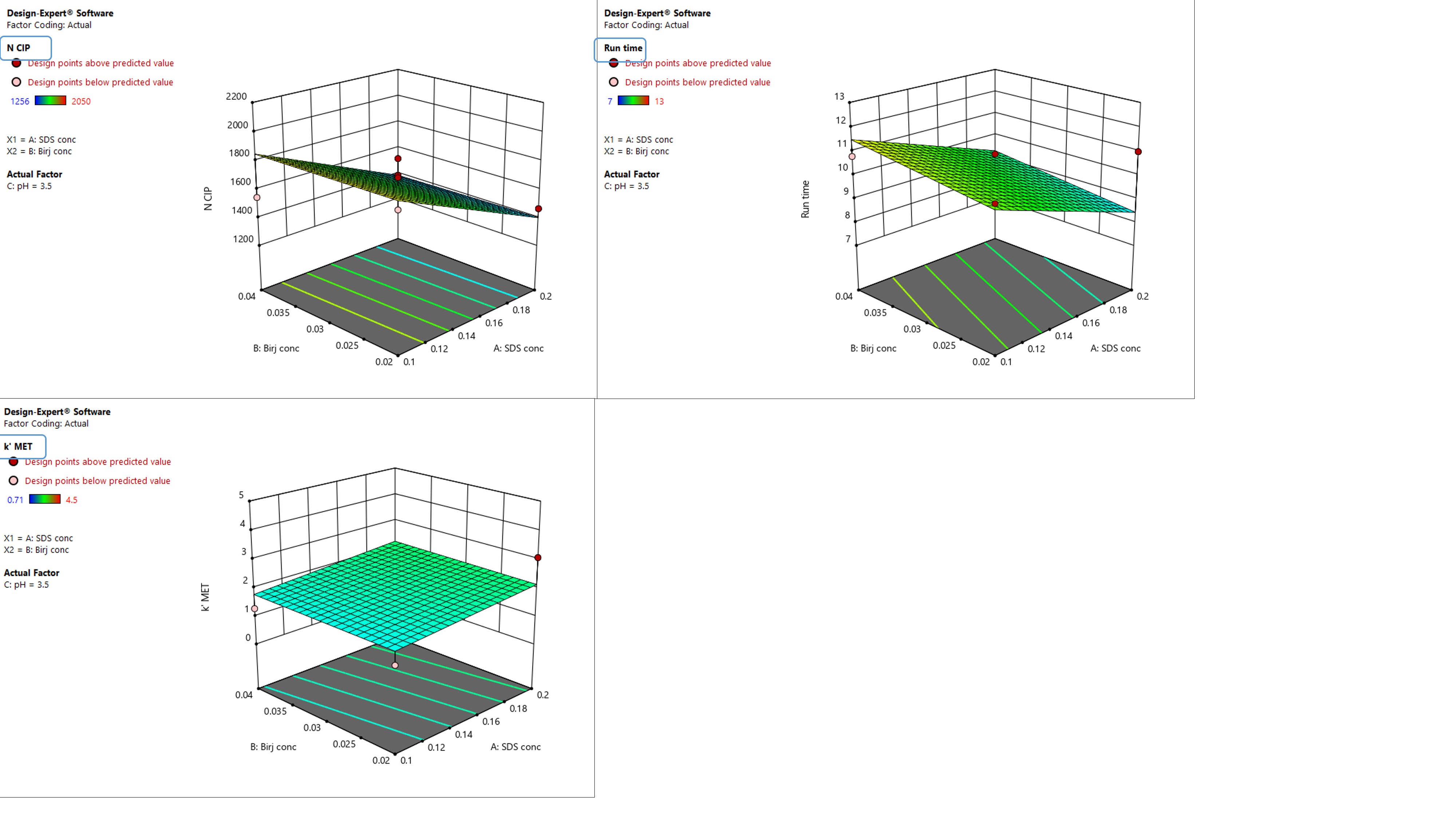


**Supplementary Fig. S2**


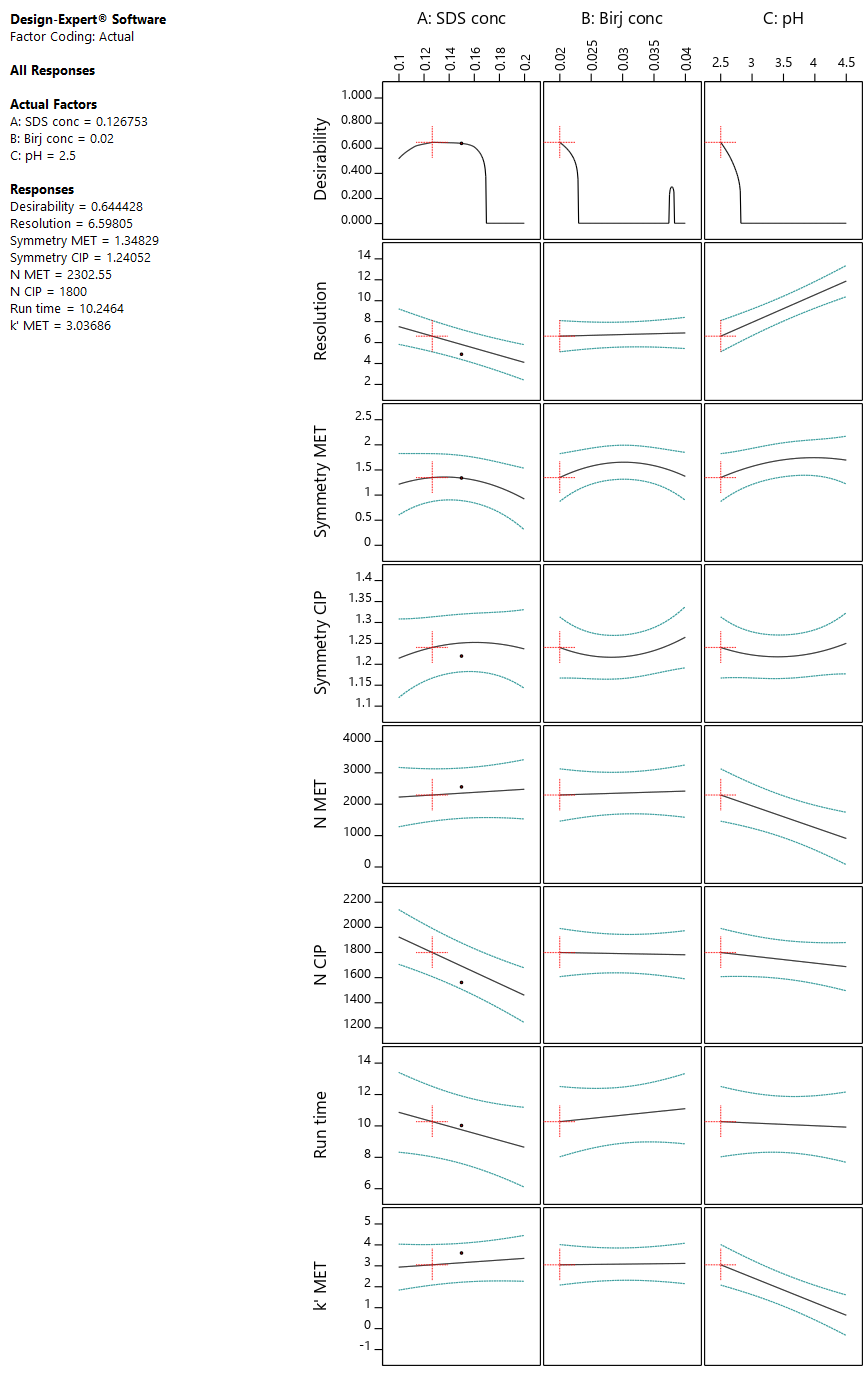


**Supplementary Fig. S3**


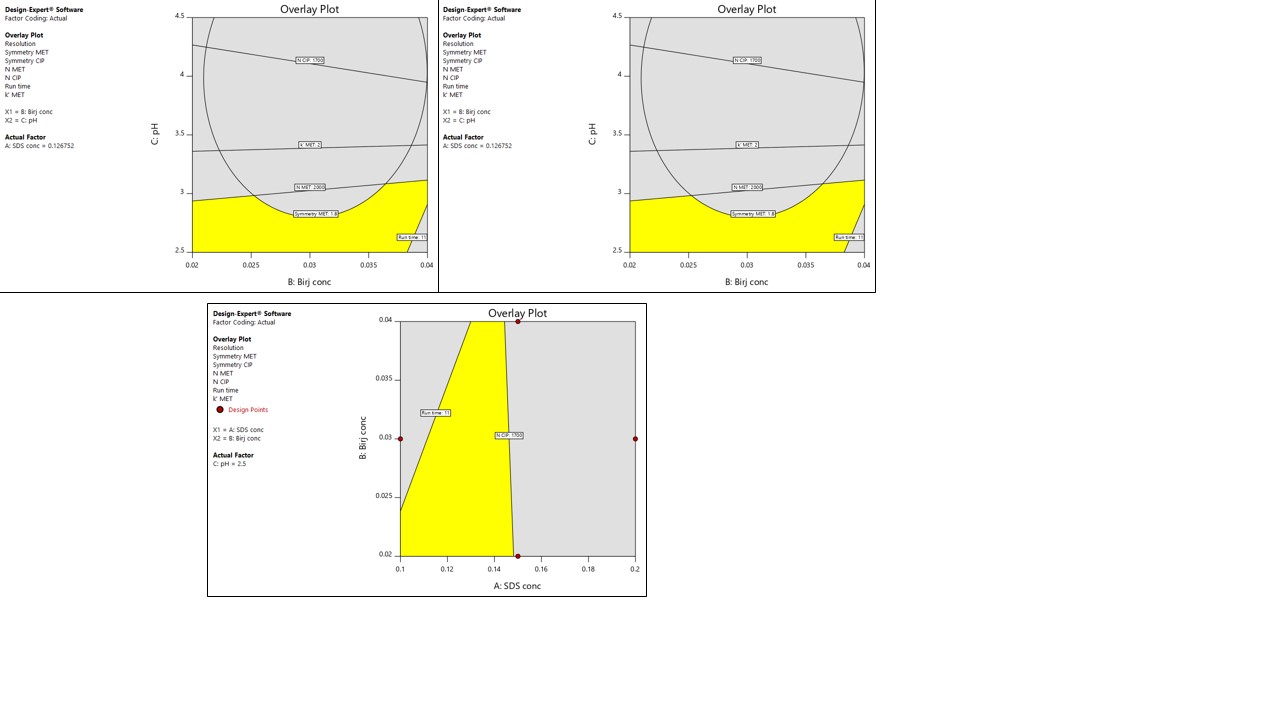


**Supplementary Fig. S4**

**
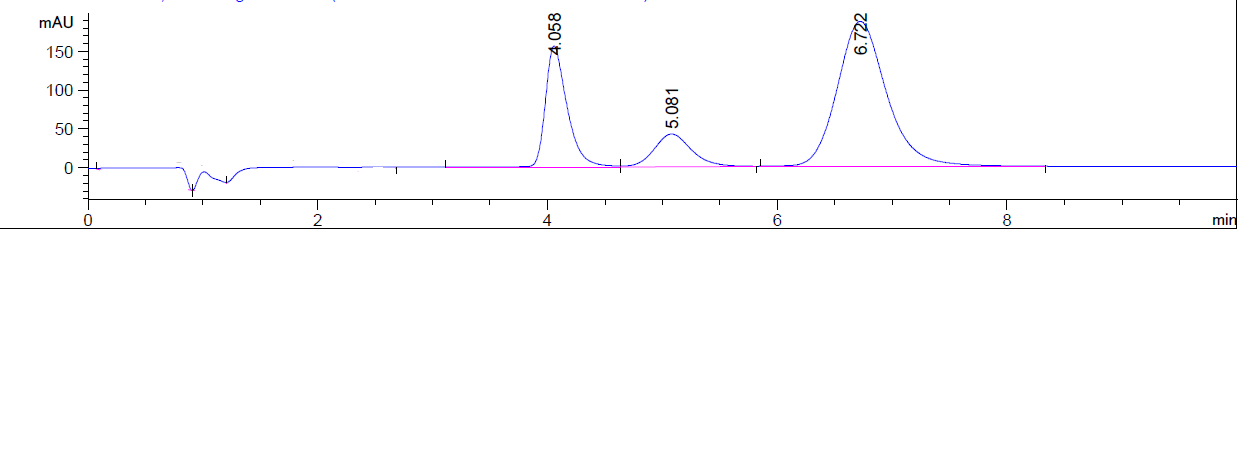
**

**Supplementary Fig. S5**

Table S1: Fractional factorial design matrix for the effect of different CMPs on CQAs

| Run | A:SDS conc (M) | B:Birj conc (M) | C:pH | D:Column Temp (°C) | E:TEA | Resolution | Tailing MTR | Tailing CIP | N MTR | N CIP | k' MTR | Run time | Ecoscale |
| --- | --- | --- | --- | --- | --- | --- | --- | --- | --- | --- | --- | --- | --- |
| 1 | 0.20 | 0.01 | 2.5 | 40 | 0 | 2.77 | 1.33 | 1.18 | 2103 | 1122 | 2.53 | 6.9 | 97 |
| 2 | 0.20 | 0.04 | 6.5 | 40 | 0 | 8.43 | 1.34 | 1.41 | 1869 | 1093 | 1.14 | 8.3 | 97 |
| 3 | 0.20 | 0.01 | 6.5 | 45 | 0 | 8.14 | 1.38 | 1.35 | 1764 | 746 | 2.21 | 17.0 | 97 |
| 4 | 0.05 | 0.01 | 6.5 | 40 | 0 | 14.69 | 1.34 | 1.33 | 1653 | 1127 | 0.52 | 22.0 | 97 |
| 5 | 0.20 | 0.04 | 2.5 | 45 | 0.1 | 4.90 | 1.35 | 1.21 | 2312 | 1510 | 2.17 | 6.9 | 91 |
| 6 | 0.05 | 0.01 | 6.5 | 45 | 0 | 14.73 | 1.37 | 1.35 | 1619 | 1382 | 0.44 | 18.2 | 97 |
| 7 | 0.05 | 0.04 | 6.5 | 45 | 0.1 | 7.02 | 1.34 | 1.42 | 1725 | 751 | 0.46 | 15.0 | 91 |
| 8 | 0.20 | 0.01 | 6.5 | 40 | 0.1 | 9.22 | 1.35 | 1.22 | 1655 | 1154 | 0.52 | 7.2 | 91 |
| 9 | 0.20 | 0.04 | 2.5 | 40 | 0.1 | 4.56 | 1.36 | 1.21 | 2307 | 1393 | 2.40 | 7.1 | 91 |
| 10 | 0.05 | 0.04 | 2.5 | 45 | 0 | 9.11 | 1.47 | 1.26 | 1633 | 1491 | 2.36 | 12.5 | 97 |
| 11 | 0.05 | 0.04 | 2.5 | 40 | 0.1 | 9.81 | 1.22 | 1.24 | 2935 | 1562 | 2.47 | 12.3 | 91 |
| 12 | 0.05 | 0.01 | 2.5 | 45 | 0.1 | 9.56 | 1.19 | 1.26 | 2834 | 1815 | 5.24 | 21.0 | 91 |

N: Number of theoretical plates

k': capacity factor

**Table S2:** Box Behnken design matrix for the effect of different CMPs on CQAs

| Run | A:SDS conc (M) | B:Birj conc (M) | C:pH | Resolution | Tailing MTR | Tailing CIP | N MTR | N CIP | Run time | k' MTR |
| --- | --- | --- | --- | --- | --- | --- | --- | --- | --- | --- |
| 1 | 0.10 | 0.03 | 2.5 | 6.99 | 1.35 | 1.21 | 3560 | 2050 | 9.8 | 2.91 |
| 2 | 0.15 | 0.02 | 2.5 | 4.87 | 1.34 | 1.22 | 2561 | 1560 | 10.0 | 3.60 |
| 3 | 0.20 | 0.03 | 2.5 | 4.25 | 1.35 | 1.22 | 2315 | 1374 | 8.2 | 2.91 |
| 4 | 0.10 | 0.04 | 3.5 | 10.41 | 1.82 | 1.25 | 1055 | 1546 | 10.8 | 1.28 |
| 5 | 0.20 | 0.04 | 3.5 | 6.47 | 0.96 | 1.24 | 1879 | 1256 | 7.0 | 1.51 |
| 6 | 0.15 | 0.02 | 4.5 | 10.81 | 1.55 | 1.26 | 1305 | 1536 | 8.5 | 0.71 |
| 7 | 0.15 | 0.04 | 4.5 | 11.41 | 1.64 | 1.39 | 1707 | 1706 | 12.9 | 1.63 |
| 8 | 0.10 | 0.03 | 4.5 | 11.95 | 1.87 | 1.23 | 1239 | 1819 | 10.9 | 1.15 |
| 9 | 0.10 | 0.02 | 3.5 | 10.61 | 1.80 | 1.21 | 844 | 1811 | 10.9 | 1.27 |
| 10 | 0.15 | 0.03 | 3.5 | 8.85 | 1.98 | 1.24 | 1020 | 1703 | 9.1 | 1.51 |
| 11 | 0.15 | 0.03 | 3.5 | 8.82 | 1.97 | 1.20 | 1018 | 1686 | 9.2 | 1.43 |
| 12 | 0.20 | 0.02 | 3.5 | 6.66 | 0.98 | 1.23 | 2145 | 1466 | 11.0 | 3.08 |
| 13 | 0.15 | 0.04 | 2.5 | 5.89 | 1.39 | 1.27 | 2707 | 1793 | 13.0 | 4.50 |
| 14 | 0.20 | 0.03 | 4.5 | 8.89 | 1.44 | 1.24 | 1355 | 1263 | 7.3 | 0.79 |
| 15 | 0.15 | 0.03 | 3.5 | 11.95 | 1.91 | 1.23 | 1239 | 1819 | 10.9 | 1.15 |

N: Number of theoretical plates

k': capacity factor

|  | **Resolution** | | **Tailing MTR** | | **Tailing CIP** | | **N MTR** | |
| --- | --- | --- | --- | --- | --- | --- | --- | --- |
| **Factor** | **Coefficient** | **p-value** | **Coefficient** | **p-value** | **Coefficient** | **p-value** | **Coefficient** | **p-value** |
| **Intercept** | 8.58 |  | 1.34 |  | 1.29 |  | 2034.08 |  |
| **A-SDS conc** | -2.24 | 0.0070 |  |  |  |  |  |  |
| **B-Birj conc** | -0.7600 | 0.2670 | 0.0275 | 0.2434 | 0.0281 | 0.0989 | -11.87 | 0.9085 |
| **C-pH** | 1.54 | 0.0445 | 0.0125 | 0.5808 | 0.0694 | 0.0014 | -323.87 | 0.0141 |
| **D-Column Temp** |  |  |  |  |  |  | -52.92 | 0.5909 |
| **E-TEA** |  |  | -0.0400 | 0.1064 |  |  |  |  |
| **AB** | 0.9000 | 0.1736 | -0.0117 | 0.5725 |  |  |  |  |
| **BD** |  |  |  |  |  |  | -187.25 | 0.0866 |

**Table S3:** Coefficients and p-values of FFD for method parameters affecting responses (resolution, tailing MTR and CIP, and N of MTR) for MTR and CIP chromatographic analysis

|  | **N CIP** | | **K**' **MTR** | | **Run time** | | **Eco-scale** | |
| --- | --- | --- | --- | --- | --- | --- | --- | --- |
| **Factor** | **Coefficient** | **p-value** | **Coefficient** | **p-value** | **Coefficient** | **p-value** | **Coefficient** | **p-value** |
| **Intercept** | 1262.17 |  | 1.87 |  | 12.87 |  | 96.00 |  |
| **A-SDS conc** |  |  |  |  | -3.97 | 0.0020 |  |  |
| **B-Birj conc** |  |  | -0.4144 | 0.1723 | -2.52 | 0.0238 |  |  |
| **C-pH** | -220.00 | 0.0033 | -1.13 | 0.0029 |  |  |  |  |
| **E-TEA** |  |  |  |  |  |  | -3.00 | 0.035 |
| **AB** | 158.06 | 0.0232 |  |  |  |  |  |  |
| **AC** | 100.69 | 0.1123 |  |  |  |  |  |  |

**Table S4:** Coefficients and p-values of FFD for method parameters affecting responses (N of CIP, k' of MTR, run time, and ecoscale) for MTR and CIP chromatographic analysis

**Table S5:** Robustness results for the proposed HPLC method

| CIP HCl | | | | MTR | | | | Parameters | |
| --- | --- | --- | --- | --- | --- | --- | --- | --- | --- |
| R.S.D | **S.D** | **Mean recovery %** | **Recovery %** | **R.S.D** | **S.D** | **Mean recovery %** | **Recovery %** |  |  |
| 0.73 | 0.73 | 99.54 | 100.25 | 0.92 | 0.91 | 99.15 | 98.25 | 0.125 | SDS conc (M) |
|  |  |  | 99.57 |  |  |  | 99.14 | 0.13 |  |
|  |  |  | 98.79 |  |  |  | 100.07 | 0.135 |  |
| 0.56 | 0.56 | 98.94 | 98.74 | 0.49 | 0.49 | 99.63 | 100.12 | 0.015 | Birij-35 conc (M) |
|  |  |  | 99.57 |  |  |  | 99.14 | 0.02 |  |
|  |  |  | 98.51 |  |  |  | 99.64 | 0.025 |  |
| 0.48 | 0.48 | 99.21 | 99.38 | 0.56 | 0.56 | 99.66 | 100.25 | 2.4 | pH |
|  |  |  | 99.57 |  |  |  | 99.14 | 2.5 |  |
|  |  |  | 98.67 |  |  |  | 99.60 | 2.6 |  |
| 0.25 | 0.25 | 99.83 | 100.07 | 0.42 | 0.41 | 99.03 | 98.57 | 38 | Column Temperature (°C) |
|  |  |  | 99.57 |  |  |  | 99.14 | 40 |  |
|  |  |  | 99.84 |  |  |  | 99.37 | 42 |  |

SD: standard deviation

RSD: Relative standard deviation

**Table S6:** Recovery of MTR and CIP HCl from Ciprodiazole tablet using the proposed HPLC method

| Drug | Concentration taken (µg/mL) | Mean concentration found * (µg/mL) | Mean % Recovery ± SD |
| --- | --- | --- | --- |
| MTR | 25 | 24.86 | 99.45 ± 0.72 |
| CIP HCl | 25 | 25.03 | 100.13 ± 0.81 |

* The mean of six determinations.

SD: standard deviation

Table S7: Application of the proposed method and comparison method for the determination of the studied drugs in Ciprodiazole tablet.

| Conc. Taken (µg/mL) | | Proposed method | | Reported method | |
| --- | --- | --- | --- | --- | --- |
|  |  | % Recovery | | % Recovery | |
| MTR | CIP HCl | MTR | CIP HCl | MTR | CIP HCl |
| 25 | 25 | 99.30 | 100.16 | 98.57 | 99.09 |
|  |  | 98.73 | 99.37 | 98.96 | 98.756 |
|  |  | 99.48 | 101.24 | 101.26 | 99.58 |
|  |  | 100.04 | 100.86 | 100.59 | 98.61 |
|  |  | 98.54 | 98.88 | 99.37 | 100.34 |
|  |  | 100.62 | 100.25 | 101.67 | 98.07 |
| Mean + SD | | 99.45 ± 0.72 | 100.13 ± 0.81 | 100.07+1.17 | 99.07+0.73 |
| t-test | | 1.86 (2.228)* | | 1.81 (2.228)* | |
| F-test | | 2.66 (5.050)* | | 1.23 (5.05)* | |

*Values in parenthesis are the tabulated t- and F- values at p=0.05.

**Table S8:** Calculation of Analytical Eco-Scale for the developed HPLC method

| **Reagents/Instruments** | **Penalty points** |
| --- | --- |
| **Reagents** | |
| Phosphoric acid | 2 |
| SDS | 0 |
| Birij-35 | 0 |
| **Instruments** | |
| HPLC | 1 |
| Occupational hazards | 0 |
| Waste | 0 |
| **Total penalty points** | ∑ 3 |
| **Analytical Eco-Scale score** | 97 |
